# Serological and Molecular Investigation of SARS-CoV-2 in Horses and Cattle in Switzerland from 2020 to 2022

**DOI:** 10.3390/v16020224

**Published:** 2024-01-31

**Authors:** Julia Hüttl, Katja Reitt, Marina L. Meli, Theres Meili, Eva Bönzli, Benita Pineroli, Julia Ginders, Angelika Schoster, Sarah Jones, Grace B. Tyson, Margaret J. Hosie, Nicola Pusterla, Kerstin Wernike, Regina Hofmann-Lehmann

**Affiliations:** 1 Center for Laboratory Medicine, Veterinary Diagnostic Services, Frohbergstrasse 3, 9001 St. Gallen, Switzerland; katja.reitt@zlmsg.ch; 2 Clinical Laboratory, Vetsuisse Faculty, Department of Clinical Diagnostics and Services, and Center for Clinical Studies, University of Zurich, Winterthurerstrasse 260, 8057 Zurich, Switzerland; mmeli@vetclinics.uzh.ch (M.L.M.); tmeili@vetclinics.uzh.ch (T.M.); eboenzli@vetclinics.uzh.ch (E.B.); bpineroli@vetclinics.uzh.ch (B.P.); jginders@vetclinics.uzh.ch (J.G.); rhofmann@vetclinics.uzh.ch (R.H.-L.); 3 Clinic for Equine Internal Medicine, Equine Department, University of Zurich, 8057 Zurich, Switzerland; angelika.schoster@lmu.de; 4 School of Biodiversity, One Health and Veterinary Medicine, College of Medical, Veterinary and Life Sciences, University of Glasgow, Bearsden Road, Glasgow G61 1QH, UK; sarah.jones.4@glasgow.ac.uk (S.J.);; 5 MRC-University of Glasgow, Centre for Virus Research, Bearsden Road, Glasgow G61 1QH, UK; margaret.hosie@glasgow.ac.uk; 6 Department of Medicine and Epidemiology, School of Veterinary Medicine, University of California, Davis, CA 95616, USA; npusterla@ucdavis.edu; 7 Institute of Diagnostic Virology, Friedrich-Loeffler-Institut (FLI), Suedufer 10, 17493 Greifswald-Insel Riems, Germany; kerstin.wernike@fli.de

**Keywords:** SARS-CoV-2, animal, horse, cattle, serology, RT-PCR, neutralization, bovine coronavirus, one health, spillover

## Abstract

Horses and cattle have shown low susceptibility to SARS-CoV-2, and there is no evidence of experimental intraspecies transmission. Nonetheless, seropositive horses in the US and seropositive cattle in Germany and Italy have been reported. The current study investigated the prevalence of antibodies against SARS-CoV-2 in horses and cattle in Switzerland. In total, 1940 serum and plasma samples from 1110 horses and 830 cattle were screened with a species-specific ELISA based on the SARS-CoV-2 receptor-binding domain (RBD) and, in the case of suspect positive results, a surrogate virus neutralization test (sVNT) was used to demonstrate the neutralizing activity of the antibodies. Further confirmation of suspect positive samples was performed using either a pseudotype-based virus neutralization assay (PVNA; horses) or an indirect immunofluorescence test (IFA; cattle). The animals were sampled between February 2020 and December 2022. Additionally, in total, 486 bronchoalveolar lavage (BAL), oropharyngeal, nasal and rectal swab samples from horses and cattle were analyzed for the presence of SARS-CoV-2 RNA via reverse transcriptase quantitative polymerase chain reaction (RT-qPCR). Six horses (0.5%; 95% CI: 0.2–1.2%) were suspect positive via RBD-ELISA, and neutralizing antibodies were detected in two of them via confirmatory sVNT and PVNA tests. In the PVNA, the highest titers were measured against the Alpha and Delta SARS-CoV-2 variants. Fifteen cattle (1.8%; 95% CI: 1.0–3.0%) were suspect positive in RBD-ELISA; 3 of them had SARS-CoV-2-specific neutralizing antibodies in sVNT and 4 of the 15 were confirmed to be positive via IFA. All tested samples were RT-qPCR-negative. The results support the hypotheses that the prevalence of SARS-CoV-2 infections in horses and cattle in Switzerland was low up to the end of 2022.

## 1. Introduction

Several animal species, ranging from wild animals like white-tailed deer to farmed or domestic animals such as minks, cats and dogs have been reported to be susceptible to infection with SARS-CoV-2, the causative agent of COVID-19 [1,2,3,4,5,6]. SARS-CoV-2 infection occurs through the binding of the viral surface glycoprotein, the spike (S) protein, to angiotensin-converting enzyme 2 (ACE2), which is widespread in several tissues among mammals [7]. ACE-2 binding affinity to SARS-CoV-2 has been investigated in 410 vertebrate species using protein structure and genomic analysis [8]. Horses were found to have a low affinity and cattle were found to have a medium affinity for SARS-CoV-2 compared to humans [8]. In an antibody analysis, the presence of ACE2-expressing cells was detected in the tracheal and bronchiolar epithelia of sheep and cattle [9,10]. These results indicate that horses and cattle should be considered potential secondary reservoirs of SARS-CoV-2.

So far, no COVID-19 outbreaks due to SARS-CoV-2 infection in domestic horses (*Equus ferus caballus*) have been officially reported to the World Organization for Animal Health (WOAH) [1]. Horses in contact with SARS-CoV-2-infected humans and a single experimentally infected horse showed no clinical signs or detectable shedding of the virus in nasal secretions, blood or feces via reverse transcriptase quantitative polymerase chain reaction (RT-qPCR) [11,12,13]. In a serological study in the US, 5.9% of 587 horses had antibodies against the receptor-binding domain (RBD) of SARS-CoV-2 after close contact with humans with asymptomatic SARS-CoV-2 infection [12]. Moreover, 3.5% of 1186 horses presented to a veterinary hospital in California had antibodies against SARS-CoV-2, and seasonality was shown with more seropositive animals in the spring season [14]. In these two serological studies, confirmatory virus neutralization assays were not performed, and seropositive serum samples determined via the RBD enzyme-linked immunosorbent assay (ELISA) were defined as suspect positive [12,14]. However, seroconversion has been observed in both RBD-ELISA and plaque reduction neutralization tests (PRNT) on one horse after direct contact with an owner infected with SARS-CoV-2 [13]. The significance of horses in the COVID-19 pandemic and the predisposing factors to equine COVID-19 infection remain unclear and require more investigation.

According to current knowledge, ruminants play a more important role in the epidemiology of SARS-CoV-2 than do equines, as multiple cases of SARS-CoV-2 infections in white-tailed (*Odocoileus virginianus*) and mule deer (*Odocoileus hemionus*) populations in North America have been reported to the WOAH [1]. In addition, 109 independent spillover events from humans to white-tailed deer were identified [4], and neutralizing antibodies against SARS-CoV-2 were detected in 40% of wild white-tailed deer tested in the US [15]. This suggests that white-tailed deer are highly susceptible to SARS-CoV-2 infection and can even transmit the virus through direct contact as well as vertically from doe to fetus [16]. In domestic cattle (*Bos taurus*), several experimental infections have been reported. Experimentally infected calves have tested positive for viral RNA in nasal swabs two to three days after inoculation with SARS-CoV-2, and visible, although incomplete, inhibition in the virus neutralization test was detected [17]. In another experimental study, no infectious viral shedding in calves was observed, although viral RNA was detected in the trachea upon necropsy [11]. Moreover, three naturally occurring SARS-CoV-2 infection outbreaks in domestic cattle from Germany have been recorded in a global open-access dataset of SARS-CoV-2 events in animals [2]. A serological screening study from Germany in 2021 reported 11 out of 1000 cattle samples to be positive via RBD-ELISA, and all but 1 of them could be confirmed via an indirect immunofluorescence assay, (IFA) while 4 samples also tested positive in the sVNT [18]. In addition, in a serological study from Italy, 13 lactating cows with neutralizing antibodies against SARS-CoV-2 were found on a farm with farm workers with COVID-19-related symptoms [19]. However, to date, no clinical signs of SARS-CoV-2 infection have been reported in seropositive cattle [17,19].

The close contact between horses and humans, as well as the high stocking densities of livestock animals, could increase the risk of virus transmissions, mutations and even recombination with other coronaviruses; therefore, it is important to monitor the susceptibility of horses and cattle to SARS-CoV-2 [20]. In this study, we investigated the seroprevalence of SARS-CoV-2 and the prevalence of RT-qPCR-detected SARS-CoV-2 in horses and cattle from Switzerland. Our aims were to evaluate the animals’ susceptibility under natural conditions and to investigate the potential for SARS-CoV-2 spillover to these species.

## 2. Materials and Methods

### 2.1. Study Population and Sample Collection

For the serological analyses, serum and plasma samples from 1110 horses, which were collected from 25 February 2020 to 28 December 2022, were included. Samples from 830 cattle were collected from 25 February 2020 to 30 May 2022.

For the molecular analyses, 244 bronchoalveolar lavage (BAL) samples from horses were included that had been collected from 30 April 2020 to 10 November 2022, and two nasal swabs from cattle were collected in September 2020. The sample material was likely to be from animals that were tested for respiratory disease.

These sample materials (serum, plasma and BAL) consisted of residual material from samples submitted to the diagnostic laboratory (Clinical Laboratory, Vetsuisse Faculty, University of Zürich) for routine diagnostic purposes. No additional samples or volumes were collected for this study. The samples were stored in the Vetsuisse Biobank at −80 °C until testing. The samples were pseudonymized; thus, no information was available concerning the potential contact of the animals to COVID-19-affected owners or animal caretakers.

For molecular analyses, samples from horses and cattle were collected prospectively, independent of clinical signs or history of COVID-19 contact, from equine and bovine patients at the Equine and Farm Animal Clinics of the University Animal Hospital Zurich between 28 April 2020 and 29 November 2020 (240 samples for RT-qPCR from 67 horses and 14 cattle). The sample collection for active recruitment was officially approved by the ethical committee of the canton of Zurich (BASEC number 2020–00979) and by the veterinary office of the canton of Zurich (ZH062/20). Oropharyngeal (*n* = 81), nasal (*n* = 81) and rectal (*n* = 78) swab samples were collected by veterinarians or veterinary staff during the clinical examination in accordance with a given sample collection protocol and as previously described [21]. Prior to sample collection, the owners were informed about the study and their written consent was obtained.

### 2.2. SARS-CoV-2 Immunoassays

#### 2.2.1. Enzyme-Linked Immunosorbent Assay (ELISA)

All serum samples were first screened using an in-house established ELISA to detect antibodies binding to the SARS-CoV-2 spike glycoprotein receptor-binding domain (RBD), as previously described for cats and dogs [21], with species-specific modifications to determine suspect positive samples. Suspect positive samples were later validated with confirmatory tests. Briefly, 96-well microtiter plates were coated with 200 ng of antigen/well using recombinant SARS-CoV-2 Spike Protein RBD, Wuhan-Hu-1 (LU2020, LubioScience GmbH, Zurich, Switzerland). Plates were then covered and incubated for 3 h at 37 °C and overnight at 4 °C. Diluted (1:100) controls and serum or plasma samples were then pipetted to each plate in a total volume of 100 µL/well; each sample was run in duplicate. All sera were previously heat-inactivated at 56 °C for 1 h. Depending on the investigated species, either a rabbit anti-horse IgG horseradish peroxidase (HRP)-conjugated secondary antibody (Jackson ImmunoResearch Europe, Ely, UK) or a goat anti-bovine IgG HRP-conjugated secondary antibody (Jackson ImmunoResearch) was added. The conjugates were diluted at 1:3000, and 100 µL/well was used. A substrate solution containing ABTS (2.2-azino-di (3-ethylbenzthiazoline-6-sulfonic acid diammonium salt)) (Sigma-Aldrich Chemie GmbH, Buchs, Switzerland) was pipetted into each well, and then optical density (OD) was measured immediately afterward for horse samples and after 10 min for cattle samples at 415 nm in a microplate photometer (SpectraMax Plus 384, Molecular Devices LLC., San Jose, CA, USA). The standardized OD was calculated as follows: SOD = (OD value [sample] − OD value [negative control])/(OD value [positive control] − OD value [negative control]).

Serum samples from five SARS-CoV-2 antibody-positive horses were provided by Dr. Nicola Pusterla, School of Veterinary Medicine, University of California, Davis, CA, USA. The samples were taken from healthy adult racing Thoroughbreds (#850, 859, 912, 932 and 1020) for a screening study [12]. We used one of these samples (#912) as the positive control in the RBD-ELISA (standardized OD 1.0). The cut-off value for suspect positive horse samples in the RBD-ELISA was set at four standard deviations above the mean value of the SOD of 24 samples from a pre-COVID-19 cohort of horses collected in Switzerland in 2014 (residual material from samples submitted to the Clinical Laboratory).

The positive control serum for cattle used in the RBD-ELISA originated from a study of experimentally infected cows [17] and was provided by Dr. Lorenz Ulrich and Dr. Kerstin Wernike, Friedrich-Loeffler-Institut, Greifswald-Insel Riems, Germany. The sample was collected from cow number 776 at 20 days after inoculation with SARS-CoV-2 [17]. The cut-off value for suspect positive cattle samples was determined as four standard deviations above the mean value of the SOD of 44 samples from a pre-COVID-19 cohort of cattle collected in Switzerland in 2004 (residual material from samples submitted to the Clinical Laboratory).

#### 2.2.2. Surrogate Virus Neutralization Test

The commercially available surrogate virus neutralization test (SARS-CoV-2 Surrogate Virus Neutralization Test (sVNT) Kit, GenScript, Rijswijk, the Netherlands) was performed for samples with suspect positive results from RBD-ELISA. The test allows the detection of neutralizing antibodies; if present, they will block the interaction between the RBD of the SARS-CoV-2 spike glycoprotein and the ACE2 cell surface receptor [22]. It was performed in accordance with the manufacturer’s instructions. Optical density was measured at 450 nm, and the percentage of inhibition for each sample was calculated using the following formula: % inhibition = (1 − (OD450 sample/OD450 of negative control)) × 100. Controls were run in duplicate, and samples were analyzed once.

The commercial sVNT guidelines include a cut-off of 30% for human samples, but for animal samples, no recommended cut-off values are available. Nevertheless, the sVNT demonstrated moderate to high sensitivity and high specificity when evaluated with sera from nine animal species, including cattle [22]. However, more elaborate species-specific validation would be required due to species-dependent differences in the sensitivity of the test [22]. Pre-COVID-19 samples from 24 horses and 20 of the 44 cattle (see above) were tested to determine cut-off values for both species in sVNT. For both horse and cattle samples, the cut-off was set as four standard deviations above the mean value of reactivity of their respective pre-COVID-19 cohorts.

#### 2.2.3. Pseudotype-Based Virus Neutralization Assay

For further confirmatory testing, the horse samples with suspect positive results in the RBD-ELISA were sent to the MRC-University of Glasgow Centre for Virus Research for pseudotype-based virus neutralization assays (PVNA), which have been previously described [23]. HIV (SARS-CoV-2) pseudotypes were prepared that expressed a luciferase gene and either B.1, Alpha, Delta, Omicron BA.1 or BA.2 spike proteins. First, samples were incubated for one hour with each pseudotype at a single sample dilution of 1:50. HEK293-ACE2 cells were then added, and 48–72 h incubation was performed. Luciferase activity was then measured. If samples contained neutralizing antibodies, luciferase activity was reduced, as the pseudotypes were prevented from entering the cells. A 90% reduction in infectivity, compared to that in a no-serum control, was considered a positive result. For samples with a positive result for at least one pseudotype, neutralizing antibody titers were obtained by repeating the assay with serially diluted samples. The titer was defined as the dilution factor, which reduced the infectivity by 90%, in comparison to that of a no-serum control.

#### 2.2.4. Indirect Immunofluorescence Test

Confirmatory testing of the cattle samples with suspect positive results in the RBD-ELISA was conducted at the Friedrich-Loeffler-Institut, Greifswald-Insel Riems, Germany. Confirmation was performed by using an indirect immunofluorescence test (IFA). Titers higher than 8 in IFA were considered positive, as previously described [18].

#### 2.2.5. Assessment of Cross-Reactivity to Bovine Coronavirus

To assess potential cross-reactivity with antibodies against the bovine coronavirus (BCoV), which, like SARS-CoV-2, is classified as a betacoronavirus, two samples with antibodies against BCoV were tested via RBD-ELISA and sVNT. The two BCoV-positive serum samples were provided by Dr. Nicola Decaro, Department of Veterinary Medicine, University of Bari.

### 2.3. Molecular Analysis

To detect SARS-CoV-2 RNA, all total nucleic acid extractions were carried out using either a MagNA Pure 96 instrument with MagNA Pure 96 DNA and Viral NA Small Volume Kit or a MagNA Pure LC 2.0 instrument with either MagNA Pure LC Total Nucleic Acid Isolation Kit or MagNA Pure LC Total Nucleic Acid High Performance Kit (Roche Diagnostics AG, Rotkreuz, Switzerland), in accordance with the manufacturer’s instructions. Alternatively, for some of the samples, viral RNA was isolated using QIAamp^®^ Viral RNA Mini Kit (Qiagen, Hilden Germany). For each batch of extractions, a negative control (phosphate-buffered saline (PBS) without Ca2+ and Mg2+, Life Technologies Ltd., Paisley, UK) was included to monitor for cross-contamination. For all extractions, an input volume of 200 μL (140 μL for QIAamp Kit) was used. The extraction of oropharyngeal, nasal and rectal swabs was performed as described previously [21]. All nucleic acid samples underwent RT-qPCR targeting the viral envelope (E) and RNA-dependent RNA polymerase (RdRp) genes, as described previously [24]. Negative RT-qPCR controls (RNAse–DNase-free water, AppliChem, Darmstadt, Germany), a negative extraction control (PBS) and a positive RT-qPCR control (in vitro-transcribed RNA control containing three concatenated sequences of RdRp, E, and nucleocapsid (N) SARS-CoV-2 genes: RNA_Wuhan_RdRp-E-N) were assayed with every run. Absence of inhibition was verified by testing neat and 1:5 diluted nucleic acids for all samples. The presence of amplifiable RNA was verified from all BAL samples by testing for the 18S rRNA housekeeping gene as previously described [25].

### 2.4. Statistics

Confidence intervals for sample prevalence (CI) were calculated using GraphPad Prism Version 9 for Windows (GraphPad Prism Software LLC, San Diego, CA, USA). Data obtained from the different groups were displayed using the box plot method.

## 3. Results

### 3.1. SARS-CoV-2 RBD-ELISA

**Figure 1:**
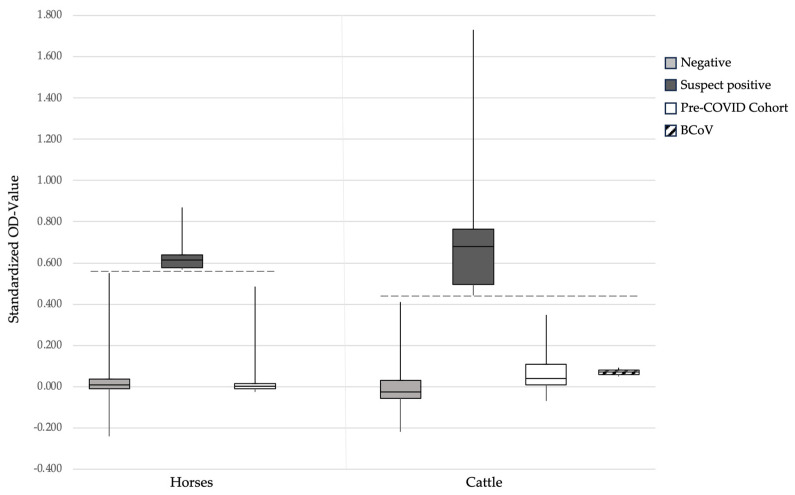
SARS-CoV-2 serological results for horse and cattle samples tested with the RBD-ELISA. For the horses (left side of the panel), the following groups are shown: 1104 Swiss samples considered negative (grey box); 6 Swiss samples considered suspect positive (standardized OD ≥ 0.568; black box); 24 negative pre-COVID-19 horse samples (white box). The cut-off for these is OD 0.568 (dashed line). For the cattle (right side of the panel), the following groups are shown: 815 Swiss samples considered negative (grey box); 15 Swiss samples considered suspect positive (standardized OD ≥ 0.431; black box); 44 negative pre-COVID-19 cattle samples (white box); 2 samples positive for antibodies against the bovine coronavirus (BCoV; diagonally striped box). The cut-off for these is OD 0.431 (dashed line). The data are shown as box plots; the boxes extend from the 25th to 75th percentiles. The horizontal line represents the median, and the whiskers extend from the smallest to the largest value.

One serum sample of a positive Californian horse (#912) was used as the positive control in the RBD-ELISA (per the definition with a standardized OD of 1.0). The standardized OD for the 24 Swiss pre-COVID-19 horse samples ranged from −0.025 to 0.483 (median 0.003; Figure 1). The cut-off OD value for suspect positive horses was calculated to be ≥0.568. This ELISA was then applied to identify suspect SARS-CoV-2 seropositive Swiss horses. The standardized OD values in the RBD-ELISA of the 1110 Swiss horse serum samples collected within this study from 2020 to 2022 ranged from −0.239 to 0.868 (median 0.010). Six samples (0.5%; 95% CI: 0.2–1.2%) were considered suspect seropositive for SARS-CoV-2 via ELISA with standardized OD values between 0.571 and 0.868 (median 0.614; Figure 1 and Table 1: #61, 199, 451, 705, 844 and 948). The remaining five samples from Californian horses were also suspect positive in the RBD-ELISA (OD ≥ 0.568), with standardized OD values between 0.875 and 1.097 (Table 1).

For the cattle samples, the serum sample from an experimentally infected cow was used as the positive control in the RBD-ELISA (per the definition with a standardized OD of 1.0). The standardized OD for the 44 Swiss pre-COVID-19 cattle samples ranged from −0.070 to 0.344 (median 0.039; Figure 1). The cut-off OD value for the suspect positive cattle was calculated to be ≥0.431. The standardized OD values in RBD-ELISA for the 830 Swiss cattle serum samples collected within this study from 2020 to 2022 ranged from 0.219 to 1.729 (median −0.024). In total, 15/830 serum samples (1.8%; 95% CI: 1.0–3.0%) were considered suspect seropositive for SARS-CoV-2 via ELISA with standardized OD values between 0.440 and 1.729 (median 0.678; Figure 1 and Table 2).

### 3.2. Surrogate Virus Neutralization Test

**Figure 2:**
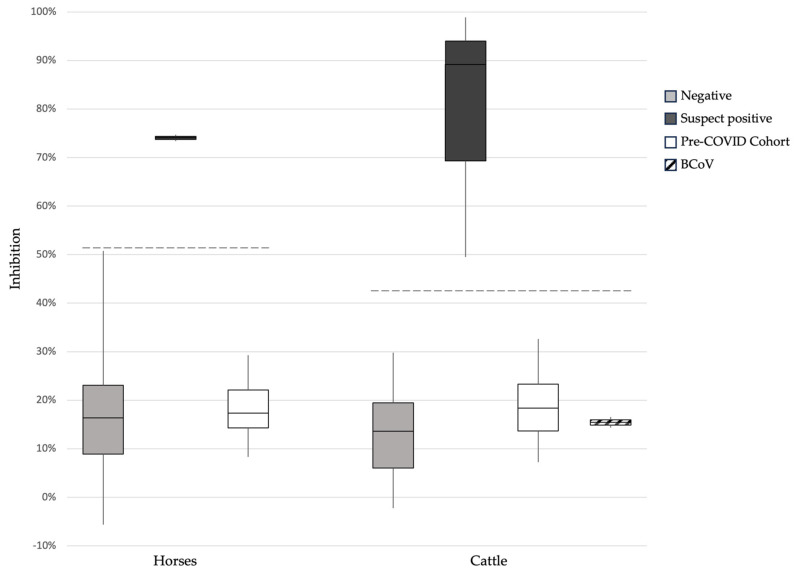
SARS-CoV-2 serological results for horse and cattle samples tested with the sVNT. For the horses (left side of the panel), the following groups are shown: 35 Swiss samples considered negative (grey box); 2 Swiss samples considered positive (>51% inhibition; black box); 24 negative pre-COVID-19 horse samples (white box). The cut-off for these is 51% (dashed line). For the cattle (right side of the panel), the following groups are shown: 17 Swiss samples considered negative (grey box); 15 Swiss samples considered suspect positive (>43% inhibition); 20 negative pre-COVID-19 cattle samples (white box); 2 samples positive for antibodies against the bovine coronavirus (BCoV; diagonally striped box). The cut-off for these is 43% (dashed line). The data are shown as box plots; the boxes extend from the 25th to 75th percentiles. The horizontal line represents the median, and the whiskers extend from the smallest to the largest value.

For the 24 Swiss pre-COVID-19 horse samples, inhibition in the sVNT ranged from 8.3% to 46.6% (median 17.7%; Figure 2). The cut-off for horses was calculated to be >51%. The 6 RBD-ELISA-suspect positive horse samples plus 31 samples with a standardized OD < 0.568 were then tested for neutralizing activity via the sVNT. Two of the RBD-ELISA-suspect positive samples were also positive in the sVNT with 74.7% (horse 844) and 73.4% inhibition (horse 948) (Table 1). The remaining four suspect positive horses (#61, 199, 451 and 705) and the 31 RBD-ELISA-negative samples were considered negative in the sVNT, with results ranging from 11.4% to 50.8% inhibition. All five samples from the Californian horses (#850, 859, 912, 932 and 1020) were positive in sVNT, with inhibition between 87.8% and 100.8%.

For the 20 Swiss pre-COVID-19 cattle samples, inhibition in the sVNT ranged from 7.2% to 32.6% (median 18.4; Figure 2). The cut-off for cattle was calculated to be >43%. In the sVNT, 3 of 15 suspect positive cattle samples in RBD-ELISA tested positive with 49.5% to 98.9% inhibition (cattle 597, 644 and 700, Table 2). The results of the remaining suspect positive and five RBD-ELISA negative samples with a standardized OD < 0.431 ranged between −2.2% and 29.8% inhibition and were considered negative in the sVNT (Figure 2). The positive control sample (cow 776) was positive in sVNT with 72.2% inhibition.

### 3.3. Pseudotype-Based Neutralization Assay

In the PVNA, two of the six horses that were suspect positive showed neutralizing activity. One of them (horse 844) had its highest neutralization titer of 168 against the Alpha variant and the other (horse 948) had its highest titer of 153 against the Delta variant (Table 1). These were the same animals that also tested positive in the sVNT assay. The remaining 4 horses (horses 61, 199, 451 and 705) that were suspect seropositive in RBD-ELISA and 18 RBD-ELISA negative horses with a standardized OD < 0.568 were considered negative in the PVNA, with neutralization titers lower than 50. No study or control samples had the highest titer against Omicron BA.1 or BA.2 (Table 1).

**Figure 3:**
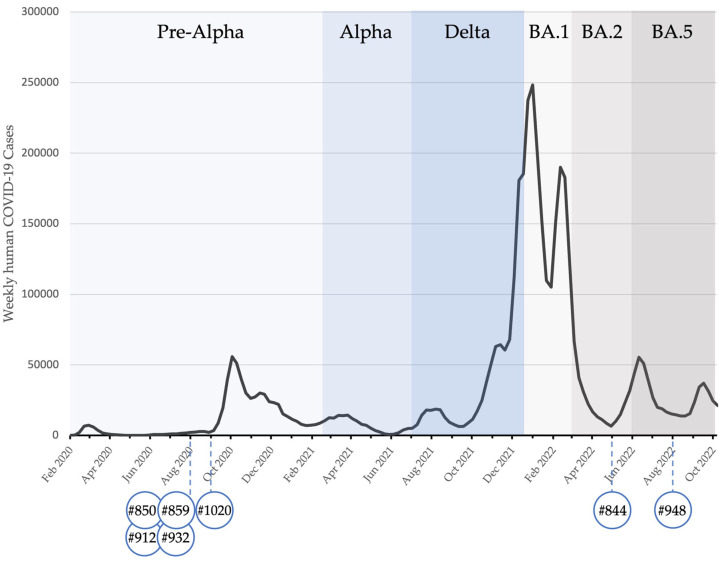
The chart shows positive horse samples with neutralizing antibodies and human SARS-CoV-2 sequences identified in Switzerland with different variants over time; human data were obtained via the opendata.swiss portal for Swiss open governmental data.

A PVNA was additionally performed on serum samples from the Californian horses (#850, 859, 912, 932 and 1020). The highest titers, which ranged from 243 to 1151, were against the B.1 variant (Table 1). When compared to the variants in humans, the highest titers against the B.1 variant mirrored its appearance in humans. The highest titers against the Alpha (horse 844) and Delta (horse 948) variants were detected after their peaks in the human cases (Figure 3).

### 3.4. Immunofluorescence Test

All 15 suspect positive bovine samples and 2 samples with a standardized OD > 0.431 were analyzed via the IFA. Four samples tested positive in the IFA with titers ranging from 8 to 128. Three of these samples were positive in both sVNT and IFA (Table 2).

### 3.5. Assessment of Potential Cross-Reactivity between SARS-CoV-2 and the Bovine Coronavirus in RBD-ELISA and sVNT

The BCoV-seropositive serum samples were negative in the RBD-ELISA with standardized OD values of 0.005 and 0.009 (Figure 1; cut-off ≥ 0.431) and negative in the sVNT with results below 16.5% (cut-off > 43%; Figure 2).

### 3.6. Molecular Analysis

In the molecular analysis of biobank samples, all 244 BAL samples from horses and 2 nasal swabs from cattle tested negative for SARS-CoV-2 RNA in RT-qPCR (Table 2). Forty-five BAL samples were assessed for sufficient DNA and the absence of PCR inhibition using the eukaryotic 18s rRNA RT-qPCR assay; all samples yielded good CT-values (range: 16–36), indicating that the quality was sufficient, and that no inhibition was present. Moreover, in the prospective sample collection, all 240 samples (81 oropharyngeal swabs, 81 nasal swabs and 78 fecal swabs or fecal samples) were negative in the SARS-CoV-2 RT-qPCR.

### 3.7. Summary of All Results

Table 3 summarizes the serological and molecular results of all samples.

## 4. Discussion

This is the first study to investigate SARS-CoV-2 infection in horses and cattle during the COVID-19 pandemic in Switzerland. Higher seroprevalence was found in cattle than that in horses. Moreover, we demonstrate neutralizing activity against different SARS-CoV-2 variants (B.1, Alpha and Delta) in horses as well as high neutralizing activity (sVNT) against SARS-CoV-2 in cattle.

Despite the expected low susceptibility of equines, we were able to detect antibodies against SARS-CoV-2 in 6 of the 1100 horses that were tested (0.5%; 95% CI 0.1–1.2%), presumably after natural infection. The six samples were suspect positive via RBD-ELISA, and two of them were assumed to also have neutralizing activity (positive sVNT and PVNA). The 1100 horses under investigation were presented to the University Animal Hospital for various reasons between February 2020 and December 2022. The prevalence we found in Swiss horses was significantly lower than what was reported in University Teaching Hospital in California, where 42 of 1186 (3.5%; 95% CI: 2.6–4.8) horses tested suspect positive between February 2020 and March 2022 [14]. Even higher prevalence was reported in racing Thoroughbred horses in California, where 35 of 587 animals (5.9%; 95% CI: 4.2–8.2%) were suspect positive; however, these horses were tested after known exposure to humans with SARS-CoV-2 infection [12]. Apart from the reported exposure in the latter population, cultural differences in horse husbandry between the two countries may act as a predisposing factor for SARS-CoV-2 infection, thereby explaining the difference in prevalence observed in American horses compared to that in Swiss horses. This may include the frequency and duration of interactions between humans and horses, for example. Moreover, differences in the screening assay could have contributed to the lower prevalence in Switzerland studies compared to that in the California studies [12,14]).

We tested five horses from the US that were sent to us as positive controls and that subsequently tested positive in the present study for neutralizing activity using the sVNT and PVNA for the B.1-, Alpha-, Delta and Omicron BA.1 variants. Thus, it appears that our cut-off for the RBD-ELISA, determined using samples from 24 Swiss pre-COVID-19 horses, might have been too restrictive, and the effective number of seropositive horses might have been higher than 0.5% in Switzerland. The setting of cut-offs for various species in this type of study is challenging. However, we had tested not only suspect positive but also RBD-ELISA-negative horse samples using the sVNT and PVNA, and those results were also negative. This further supports an acceptable to good cut-off setting for horses in the present study.

Fifteen cattle samples (1.8%; 95% CI: 1.0–3.0) were suspect positive via RBD-ELISA, and three of these had also neutralizing antibodies as determined by sVNT. The detection of antibodies against SARS-CoV-2 via RBD-ELISA was further confirmed via the IFA in four animals (0.5%; 95% CI 0.1–1.2%). In a previous study that used the same IFA to analyze residual material from routine diagnostic samples from cattle in Germany, 10 out of 1000 samples (1%; 95% CI 0.5–1.8%) were positive [11]. Thus, similar prevalence was found in Switzerland and Germany. We did not observe any cross-reactivity of RBD-ELISA or the sVNT with BCoV; this is consistent with other studies that failed to show the cross-reactivity of BCoV infection with SARS-CoV-2 [8,9]. The bovine sample in our study with the highest level of antibodies detected in RBD-ELISA was taken in June 2021 and demonstrated very high virus neutralization activity in sVNT (98.9%). This is the first time natural infection, with high (>90%) virus neutralization activity in the sVNT, has been reported in cattle. These high values suggest that the sample was taken at the peak titer of antibody levels. At the time the sample was collected, the Delta variant accounted for the majority of human COVID-19 cases in Switzerland [26].

Four horse and twelve cattle samples were suspect positive for RBD binding antibodies (ELISA) but were negative in the sVNT and/or PVNA. Thus, these results could have been potentially false positive RBD-ELISA results caused by a cross-reaction with other betacoronaviruses. While this has been tested and excluded for BCoV in the present as well as in other studies [17,27], we have not tested for equine coronavirus (ECoV), but no cross-reactivity was found for ECoV in another study [12]. Alternatively, in both the horse and cattle samples, the RBD-ELISA could have been truly positive, but the animals had not (yet) developed significant neutralizing activity. The sVNT assay used herein has been reported to be highly specific but only moderately sensitive for animal samples, since it has less sensitivity for detecting low neutralizing antibody levels [22]. The latter is a disadvantage when the timepoint of possible infection is unknown. Experimentally infected cattle have shown seroconversion via RBD-ELISA 12 days after the inoculation of SARS-CoV-2 and inhibition of viral replication in a virus neutralization test after 20 days [17]. Antibodies in horses were detected seven days after the owner’s COVID-19 diagnosis; they reached the highest titer at day 21 and remained elevated for up to 60 days when the observation was terminated [13]. It is still unknown how long antibodies against SARS-CoV-2 remain at a detectable level in horses, while antibodies against SARS-CoV-2 persist for up to ten months in dogs and cats from COVID-19-positive households [28].

In our study, Swiss horse #844 was found to have the highest PVNA antibody titers against the Alpha variant, in May 2022. In the human population of Switzerland, the Alpha variant was replaced by the Delta one as the dominant variant in June 2021 [26], approximately 11 months prior to the detection of antibodies against the Alpha variant in horse #844. The second Swiss horse, #948, had the highest PVNA antibody titers against the Delta variant of SARS-CoV-2 in September 2022, which was eight months after its last reported occurrence in humans in Switzerland (approximately in January 2022). Thus, it can be speculated that the antibodies in these two horses that neutralized the Alpha and Delta variants persisted, and remained detectable, for many months after the infection. Moreover, five SARS-CoV-2 antibody-positive horses from California (#850, 859, 912, 932 and 1020) [8] tested within the present study using the PVNA had highest antibody titers against SARS-CoV-2 B.1. It would be interesting to further determine whether or not there is a difference in the susceptibility of horses and cattle to different variants and whether or not some SARS-CoV-2 variants are more likely to infect them.

Demographic and clinical details from the positive horses and cattle (biobank samples) were unknown since the samples were pseudonymized. Without additional information or test material from other timepoints available, such as the clinical signs of the animals or a possible SARS-CoV-2 infection of the owner or animal caretakers, we could not determine the source of infection of the seropositive animals, or whether or not they shed the virus at another point during their infection.

SARS-CoV-2 RNA was not detected in any of the total 486 bronchoalveolar lavage (BAL), oropharyngeal, nasal or fecal samples from horses and cattle. The 244 BAL samples most probably originated from animals that were sampled because of clinical signs related to respiratory tract disease. So far, no clinical disease due to SARS-CoV-2 infection has been described in horses [11,12,13]. However, since horses in general, particularly sport horses, are high-performance athletes, respiratory diseases pose a serious problem, leading to performance loss. Therefore, it would be desirable to address in future studies whether or not SARS-CoV-2 infections might contribute to respiratory disease in horses. Moreover, in horses so far, no shedding of the virus has been detected, even when SARS-CoV-2 infection was proven via the seroconversion of the horses [12,13,14]. These observations in horses and the short time frame for detecting virus shedding in experimentally infected cattle speak in favor of serological testing as a method for identifying infected horses and cattle [17].

The underlying causes for the higher seroprevalence in cattle cannot be fully answered by the design of this study. It is possible that unknown prevalence factors or greater exposure pressure led to more frequent spillover from COVID-19 humans to cattle. However, in addition to this study, natural SARS-CoV-2 infections in cattle have already been reported in two different European countries, Germany and Italy [19,29]. On the other hand, there are fewer studies on SARS-CoV-2 susceptibility in horses than in cattle, and further research is needed.

## 5. Conclusions

This study shows that the prevalence of SARS-CoV-2 infection in cattle and horses in Switzerland was low from 2020 to 2022. Serological surveillance has proven to be an effective method for evaluating the SARS-CoV-2 susceptibility of different animal species and a way to detect possible reservoir hosts. Intraspecies transmission in cattle or horses has not been observed [13,17]. As we do not yet know all the potential reservoir species for SARS-CoV-2, or the susceptibility of horses and cattle to the new SARS-CoV-2 variants, it would be advisable for people with COVID-19 to avoid close contact with these animals.

## Figures and Tables

**Table 1:** Overview of antibody and neutralization results in suspect positive horses: RBD-ELISA (standardized OD); surrogate neutralization assay (sVNT; percentage of inhibition); pseudotype-based neutralization assay (PVNA): variant-specific neutralizing antibodies (titers) against B.1, Alpha, Beta, Delta and Omicron BA.1 or BA.2 variants. The first six samples (#61, 199, 451, 705, 844 and 948) originate from Swiss horses investigated in this study. The last five samples (#850, 859, 912, 932 and 1020) were from seropositive Californian horses. The cut-off OD value for a suspect positive result in RBD-ELISA is ≥0.568. The cut-off for inhibition in sVNT is >51%. The cut-off for PVNA was 50. The suspect positive RBD-ELISA results, positive sVNT results and highest titers in PVNA are marked in bold.

Sample ID	Sampling Date	RBD-ELISA	sVNT	B.1	Alpha	Delta	Omicron BA.1	Omicron BA.2
**#705**	May 2020	**0.592**	15.9	≤50	≤50	≤50	≤50	n.t.
**#199**	August 2020	**0.571**	21.8	≤50	≤50	≤50	≤50	n.t.
**#61**	December 2020	**0.640**	12.8	≤50	≤50	≤50	≤50	n.t.
**#451**	January 2022	**0.634**	11.4	≤50	≤50	≤50	≤50	n.t.
**#844**	May 2022	**0.868**	**74.7**	101	**168**	108	71	68
**#948**	August 2022	**0.566**	**73.4**	76	120	**153**	87	59
**#850**	August 2020	**0.875**	**98.2**	**243**	225	120	≤50	n.t.
**#859**	August 2020	**1.105**	**100.6**	**543**	371	207	71	n.t.
**#912**	August 2020	**1.000**	**100.4**	**363**	307	222	54	n.t.
**#932**	August 2020	**1.097**	**100.8**	**1151**	815	636	93	n.t.
**#1020**	September 2020	**0.953**	**100.3**	**568**	403	285	59	n.t.

n.t. = not tested.

**Table 2:** Overview of antibody and neutralization results from suspect positive cattle: RBD-ELISA (standardized OD); surrogate neutralization assay (sVNT; percentage of inhibition); indirect immunofluorescence test (IFA). The cut-off OD value for a suspect positive result in RBD-ELISA is ≥0.431. The cut-off for inhibition in sVNT is >43%. The cut-off for the IFA was 8. The suspect positive RBD-ELISA results, positive sVNT results and the highest titers in the IFA are marked in bold.

Sample ID	Sampling Date	RBD-ELISA	sVNT	IFA
**#184**	March 2020	**0.632**	−1.1	<8
**#89**	March 2020	**0.737**	−2.2	<8
**#825**	April 2020	**0.506**	19.6	**8**
**#700**	April 2020	**0.678**	**89.2**	**128**
**#25**	June 2020	**0.786**	18.1	<8
**#44**	June 2020	**0.583**	0.47	<8
**#2**	June 2020	**0.451**	6.0	<8
**#334**	July 2020	**0.470**	12.3	<8
**#520**	October 2020	**0.836**	19.5	<8
**#597**	June 2021	**1.729**	**98.9**	**128**
**#589**	June 2021	**0.742**	8.4	<8
**#631**	November 2021	**0.741**	11.3	<8
**#644**	December 2021	0.868	**49.5**	**16**
**#407**	April 2022	0.442	13.6	<8
**#419**	April 2022	0.483	27.5	<8

**Table 3:** Overview of the number of horse and cattle samples, which were tested for antibodies against SARS-CoV-2 (serum samples) and the presence of SARS-CoV-2 RNA (BAL, nasal, oropharyngeal and fecal samples), and the results of the tests.

	Horses No. of Samples Tested	Positive	Cattle No. of Samples Tested	Positive
RBD-ELISA	1110	6 (suspect positive)	830	15 (suspect positive)
sVNT	37	2	20	3
PVNA	24	2	n.t.	n.t.
IFA	n.t.	n.t.	17	4
BAL-PCR	244	0	n.t.	n.t.
Nasal RT-PCR	67	0	16	0
Oropharyngeal RT-PCR	67	0	14	0
Fecal RT-PCR	64	0	14	0

RBD, receptor-binding domain; sVNT, surrogate virus neutralization test; PVNA, pseudotype-based virus neutralization assay; IFA, indirect immunofluorescence test; BAL, bronchoalveolar lavage; RT-PCR, reverse transcriptase quantitative polymerase chain reaction; n.t., not tested.

## Data Availability

All available data are presented in this manuscript.
